# Relationship between perceptions of recurrence risk and depression state among first‐episode ischemic stroke patients in rural areas: The mediating role of coping style

**DOI:** 10.1002/nop2.2049

**Published:** 2024-01-06

Nursing Open. 2023 Jul;10(7):4515–4525. doi: 10.1002/nop2.1695.

In line 5 of the ‘ETHICAL APPROVAL’ section, the text ‘approved the work (ZZURIB202080)’ was incorrect. This should have read: ‘approved the work (ZZURIB202008)’.

We apologize for this error.

